# Identifying and verifying Huntington's disease subtypes: Clinical features, neuroimaging, and cytokine changes

**DOI:** 10.1002/brb3.3469

**Published:** 2024-03-17

**Authors:** Ling‐Xiao Cao, Jin‐Hui Yin, Gang Du, Qing Yang, Yue Huang

**Affiliations:** ^1^ China National Clinical Research Center for Neurological Diseases Beijing Tiantan Hospital, Capital Medical University Beijing China; ^2^ Department of Neurology Beijing Tiantan Hospital Capital Medical University Beijing China; ^3^ Department of Neurology The Third People's Hospital of Longgang District Shenzhen China; ^4^ Pharmacology Department, School of Biomedical Sciences, Faculty of Medicine and Health UNSW Sydney Sydney Australia

**Keywords:** cytokines, Huntington's disease, neuroimaging, subtype

## Abstract

**Aims:**

Huntington's disease (HD) is a progressive neurodegenerative disorder with heterogeneous clinical manifestations. Identifying distinct clinical clusters and their relevant biomarkers could elucidate the underlying disease pathophysiology.

**Methods:**

Following the Enroll‐HD program initiated in 2018.09, we have recruited 104 HD patients (including 21 premanifest) and 31 health controls at Beijing Tiantan Hospital. Principal components analysis and k‐means cluster analysis were performed to determine HD clusters. Chi‐square test, one‐way ANOVA, and covariance were used to identify features among these clusters. Furthermore, plasma cytokines levels and brain structural imaging were used as biomarkers to delineate the clinical features of each cluster.

**Results:**

Three clusters were identified. Cluster 1 demonstrated the most severe motor and nonmotor symptoms except for chorea, the lowest whole brain volume, the plasma levels of IL‐2 were higher and significantly associated with cluster 1. Cluster 2 was characterized with the most severe chorea and the largest pallidum volume. Cluster 3 had the most benign motor symptoms but moderate psychiatric problems.

**Conclusion:**

We have identified three HD clusters via clinical manifestations with distinct biomarkers. Our data shed light on better understanding about the pathophysiology of HD.

## Graphical abstract

Three clusters of patients with Huntington's disease (HD) were identified through principal components analysis (PCA). Cluster 1 demonstrated the most severe motor and nonmotor symptoms, the longest CAG repeats, the lowest whole brain volume, and higher plasma levels of IL‐2. Cluster 2 was characterized with moderate motor symptoms, the most severe chorea, and the largest pallidum volume. Cluster 3 had the most benign motor symptoms but moderate psychiatric problems.

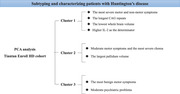

## 1 INTRODUCTION

Huntington's disease (HD) is a progressive, autosomal dominant neurodegenerative disorder, characterized by an abnormal CAG repeat expansion within the huntingtin gene (*HTT*). With an incidence of 0.38 per 100,000 individuals annually and a prevalence of 2.71 per 100,000, HD presents a significant clinical burden (Pringsheim et al., 2012). Clinically, HD is characterized by diverse motor and nonmotor symptoms, including chorea, dystonia, discordance, cognitive impairment, depression, and other behavioral difficulties (Walker, 2007). This multifaceted disease exhibits remarkable complexity and heterogeneity, as patients demonstrate variability in CAG repeats, age of onset, initial symptoms, clinical presentation, and disease progression (Rosas et al., 2008). Therefore, it is necessary to distinct HD subtypes for enhancing our understanding of the underlying pathophysiology and facilitating more targeted clinical management strategies.

Emerging evidence implicates neuroinflammation in the pathophysiology of HD (Valadao et al., 2020). The mutant huntingtin protein (mHTT) may affect astrocyte and microglia inducing inflammatory cytokines and leading to inflammation and neurodegeneration (Valadao et al., 2020). Studies have demonstrated altered cytokine profiles in the plasma of manifest HD, premanifest HD (pre‐HD), and healthy controls (HC), with correlations observed between specific cytokine levels and clinical features of HD (Bjorkqvist et al., 2008; Rocha et al., 2016). For example, the levels of tumor necrosis factor‐α (TNF‐α) in plasma correlate with chorea and the levels of interleukin‐8 (IL‐8) increase as the disease progresses (Bjorkqvist et al., 2008). These findings suggest the potential utility of cytokines as prognostic biomarkers for HD. However, investigations exploring the relationship between cytokine levels and distinct clinical subtypes of HD remain absent.

Structural magnetic resonance imaging (MRI) has shown that volume reductions can be observed in the basal ganglia, including caudate nucleus, putamen, and pallidum, in HD and even in pre‐HD (Georgiou‐Karistianis et al., 2013). This observed volumetric decline progressively intensifies with disease progression (Georgiou‐Karistianis et al., 2013). Furthermore, the whole brain and basal ganglia volumes are associated with the severity of clinical symptoms in HD (Georgiou‐Karistianis et al., 2013; Wijeratne et al., 2018), making it a sensitive and reliable biomarker. However, structural brain imaging changes has not been testified in the studies of subtyping HD (Bakels et al., 2022; Hart et al., 2013).

There are a few attempts in subtyping HD. According to the age of onset, HD can be divided into juvenile‐onset and adult‐onset HD (Bakels et al., 2022). Additionally, symptom‐based classifications proposed three subtypes: choreatic, hypokinetic‐rigid, and mixed subtypes (Hart et al., 2013). However, the arbitrary and instability of classification method limit their clinical applications. Data‐driven method disregards prior judgments about the importance of variables and uses hypothesis‐free methods to determine subtypes. The data‐driven method using principal component analysis (PCA) and k‐means cluster analysis has been suggested as the most appropriate approach and has been widely used in disease subtyping, including Parkinson's disease (PD), Alzheimer's disease (AD), multiple system atrophy (MSA), and other diseases (Fereshtehnejad et al., 2017; Habes et al., 2020; Levin et al., 2021; Mu et al., 2017; van Rooden et al., 2009; Yang et al., 2022; Young et al., 2023). This approach enables a more comprehensive integration of clinical features, including nonmotor symptoms that were previously overlooked in HD classifications. In this study, we employed a data‐driven approach to subtype HD and characterized clinical features of each subtype. Additionally, we explored neuroimaging and cytokine alterations in HD subtypes.

## 2 METHODS

### 2.1 Participants

All subjects, including manifest HD, pre‐HD, and HC, were recruited at China National Clinical Research Center for Neurological Diseases, Beijing Tiantan Hospital, from September 2018 to June 2023, following international standard Enroll‐HD protocol (https://enroll‐hd.org). All participants signed an informed consent form before enrollment. This study was approved by the Ethics Board of the Beijing Tiantan Hospital, Capital Medical University of China (KY 2018‐031‐02).

Diagnosis of HD was made by at least two senior neurologists, considering clinical manifestations, motor disturbances as assessed by the Unified Huntington's Disease Rating Scale (UHDRS) diagnostic confidence score, and the number of CAG repeats in the *HTT* gene (≥36). Pre‐HD referred to participants whose total motor score (TMS) on the UHDRS not exceeding 5 points (Bao et al., 2023). Participants with other movement disorders, such as Wilson disease and chorea‐acanthocytosis, and other neurological diseases were excluded based on genetic testing.

### 2.2 Clinical evaluation

Demographic data and clinical characteristics were obtained, including body mass index (BMI), age of onset, disease duration, family history, and number of CAG repeats. All patients were assessed for motor symptoms using UHDRS, including Motor Assessment (UHDRS‐M), Total Maximum Chorea (TMC), Functional Assessment Scale (FAS), Independence, Total Functional Capacity (TFC), as well as nonmotor symptoms by scales including Mini‐Mental State Examination (MMSE), Symbol Digit Modalities Test (SDMT), Category Fluency Test (CFT), Stroop Interference Test (SIT), Beck Depression Inventory II (BDI‐II), and Problem Behavior Assessment (PBA).

### 2.3 Inflammatory biomarkers

Plasma concentrations of cytokines were measured using V‐PLEX proinflammatory Panel 1 human kit [Meso Scale Discovery (MSD)]. The kit contains the following 10 cytokines: IL‐1β, IL‐2, IL‐4, IL‐6, IL‐8, IL‐10, IL‐12p70, IL‐13, TNF‐α, and interferon‐γ (IFN‐γ).

### 2.4 MRI data acquisition and preprocessing

Brain structural image data were acquired on a 3.0‐Tesla Siemens Prisma MRI Scanner. Foam padding and earplugs were used to limit head motion and reduce scanner noise. T1‐weighted images were acquired using a MPRAGE SAG 3D sequence with the following parameters: Voxel size: 0.9×0.9×1.0 mm; Field of View (FoV) read: 240 mm; FoV phase: 100%; Repetition time (TR): 1560 ms; Echo time (TE): 1.69 ms; Slice resolution: 100%; Flip angle: 8°; Sagittal slices: 176.

Brain structural images were preprocessed using the Computational Anatomy Toolbox 12 (CAT12) based on the Statistical Parametric Mapping 12 program (SPM12 https://www.fil.ion.ucl.ac.uk/spm/software/spm12/), which was implemented in MATLAB (http://www.mathworks.com/products/matlab/) (Gaser et al., 2006). Original DICOM format images were converted to the NIFTI format and spatially normalized. Then, the gray matter, white matter, and cerebrospinal fluid images, obtained by segmenting the MPRAGE images, were standardized to the Montreal Neurological Institute (MNI) standard space (1.5 mm × 1.5 mm × 1.5 mm). Subsequently, structural images normalized to the MNI space were registered to the Automated Anatomical Labelling Atlas 3 (AAL3) map (Rolls et al., 2020). Then, the surface and thickness estimations were performed using CAT12. We carried out quality control on the segmentation of gray matter images and excluded subjects with poor imaging quality. Finally, the volume of the bilateral caudate nucleus, putamen, globus pallidum, whole brain volume, and average cortical thickness of each subject were extracted for subsequent statistical analysis.

### 2.5 Statistical analysis

Statistical analysis was conducted using IBM SPSS 26 and R software. To avoid collinearity, PCA was performed on the motor and nonmotor symptom scales separately, after zero centering. Principal components (PCs) explaining at least 5% of the variance were retained. Cluster analysis was performed using k‐means clustering analysis. An individual was allocated to the cluster with the greatest similarity by Euclidean distance. The optimal number of clusters (k) was determined by plotting elbow plots for the total sum of squares and line chart for gap statistic (Figure S1). To evaluate differences between variables or clusters, the chi‐square test was used for the categorical variables and one‐way ANOVA for the normally distributed continuous variables. The association between cytokines and clinical characteristics was assessed by Pearson's correlation analysis. Logistic regression analysis was used to analyze the association of cytokines with each cluster. Covariance analysis was used to compare basal ganglia volumes between HD and HC, as well as differences among each cluster. Disease duration, gender, and age/age at onset were selected as covariates. A *p* value ≤ .05 was considered significant.

## 3 RESULTS

### 3.1 Clinical features of the cohort

A total 104 m*HTT* carriers, including 83 clinical manifests and 21 pre‐HD, and 31 HC participants were enrolled in this study. Demographics and clinical characteristics were shown in Table S1. The mean numbers of CAG repetitions were 46 and 44 for HD and pre‐HD, respectively. The mean disease duration for HD was 4.66 years. Subjects with HD and pre‐HD had worse performance in nonmotor symptoms than HC, and HD patients had worse performance in all motor symptoms and nonmotor symptoms than pre‐HD, assessed by UHDRS, MMSE, SDMT, CFT, SIT, BDI‐II, and PBA (Table S1).

### 3.2 Clinical evaluation of the clusters

**FIGURE 1:**
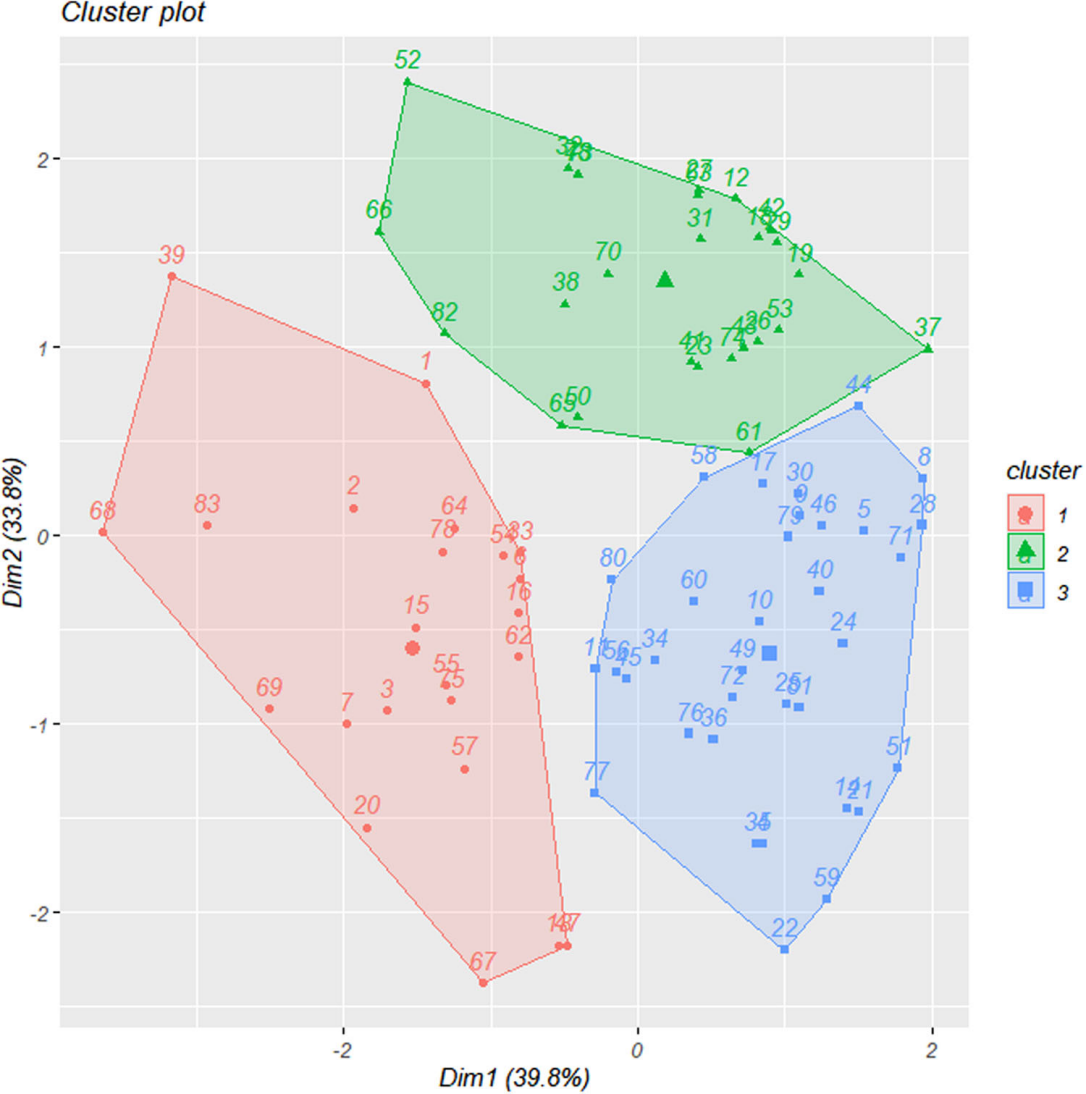
Distribution of cluster memberships through PCA analysis. Red represents cluster 1, green represents cluster 2, blue represents cluster 3. Significant differences in principal components among individuals of each cluster.

For motor PCA, the two principal components (PCs) can explain nearly 99% of the total variation. For nonmotor PCA, the two PCs can explain nearly 95% of the total variation. Cluster analysis, guided by elbow plots and gap statistics, identified three distinct clusters among the HD patients (Figure S1): cluster 1 (23 patients, 27.7%), cluster 2 (26 patients, 31.3%), and cluster 3 (34 patients, 41.0%). We found significant differences among clusters in disease duration, *HTT* CAG repetitions, motor symptoms and nonmotor symptoms (Table 1, Figure S2), as well as in PCA analysis (Figure 1). Patients in cluster 1 were clinically characterized by hypokinetic‐rigid motor deficit, and they had the highest number of *HTT* CAG repeats, the worst self‐care ability in daily life (UHDRS‐Independence), the most severe motor symptoms (UHDRS‐M) except for chorea (UHDRS‐TMC), and the most severe nonmotor symptoms including cognitive impairment (SDMT, MMSE, SIT, and CFT), depression (BDI‐II), and other mental and emotional problems (PBA) (Table 1, Figure S2). Patients with cluster 2 were clinically characterized by the most severe chorea symptom (UHDRS‐TMC) and high proportion of choreatic predominant (clinical phenotype), while the severity of overall motor symptoms (UHDRS‐M) fell between cluster 1 and cluster 3. The cognitive function of cluster 2 was better than that of cluster 1 but worse than that of cluster 3. However, depression and other mental and emotional problems in cluster 2 (BDI‐II & PBA) were the least affected among three clusters (Table 1, Figure S2). Cluster 3 is a motor‐benign phenotype, with the best performances across all UHDRS component scores and the best scores in cognition (MMSE, SDMT, SIT), but moderate mental and emotional problems assessed by BDI‐II and PBA (Table 1, Figure S2).

**TABLE 1:** Demographic and clinical characteristics of the different cluster of HD.

Clinical characteristics	Cluster 1	Cluster 2	Cluster 3	Total	*p* Value
Number (%)	23 (27.7%)	26 (31.3%)	34 (41.0%)	83 (100.0%)	NA
Male (%)	9 (39.1%)	13 (50.0%)	19 (55.9%)	41 (49.4%)	.471
Onset age, mean (SD)	39.96 (15.48) years/o	45 (6.95)	43.38 (11.77)	32.94 (11.77)	.371
Disease duration, mean (SD)	6.52 (2.50) years	4.96 (3.29)	3.18 (2.65)	4.66 (3.12)	**<.001** ^c^, ^d^
Clinical phenotype	5/12/6	18/2/6	12/14/8	35/28/10	**.004** ^a^
BMI	21.13 (3.55)	21.42 (2.43)	22.21 (2.72)	21.66 (2.89)	.344
*HTT* CAG repetitions	48.65 (11.61)	43.69 (2.38)	45.44 (4.45)	45.78 (7.05)	**.043** ^b^
Initial symptoms	16/1/6	22/2/2	30/1/3	68/4/11	.259
UHDRS‐M	67.48 (20.65)	58.92 (18.49)	25.68 (13.82)	47.67 (25.42)	**<.001** ^c^, ^d^
UHDRS‐TMC	13.87 (6.49)	15.58 (4.85)	6.09 (4.87)	11.22 (6.85)	**<.001** ^cd^
UHDRS‐FAS	12.48 (6.38)	18.31 (3.94)	22.65 (2.26)	18.47 (5.92)	**<.001** ^a^
UHDRS‐independence	61.09 (18.34)	78.85 (12.91)	92.06 (8.36)	79.34 (18.16)	**<.001** ^a^
UHDRS‐TFC	4.04 (3.04)	7.85 (3.34)	11.03 (2.20)	8.10 (4.00)	**<.001** ^a^
SIT	12.17 (4.26)	14.85 (6.60)	25.21 (9.21)	18.35 (9.29)	**<.001** ^c^, ^d^
SDMT	12.43 (6.47)	14.46 (7.43)	27.03 (7.68)	19.05 (9.86)	**<.001** ^c^, ^d^
CFT	7.70 (3.90)	7.77 (2.88)	14.12 (5.63)	10.35 (5.42)	**<.001** ^c^, ^d^
BDI‐II	20.78 (9.08)	5.81 (6.75)	10.21 (7.76)	11.76 (9.76)	**<.001** ^a^
PBA	37.78 (14.13)	5.65 (7.15)	22.12 (11.59)	21.3 (16.65)	**<.001** ^a^
MMSE	20.52 (5.04)	22.38 (6.07)	25.97 (3.26)	23.34 (5.26)	**<.001** ^c^, ^d^
Motor PC1	−88.73 (10.13)	−100.99 (7.15)	−95.62 (7.64)	−95.39 (9.43)	**<.001** ^a^
Motor PC2	27.8 (26.93)	11.21 (22.12)	−25.75 (14.99)	0.67 (31.07)	**<.001** ^a^
Nonmotor PC1	−47.55 (11.34)	−29.85 (7.76)	−52.51 (8.34)	−44.04 (13.34)	**<.001** ^a^
Nonmotor PC2	−19.41 (12.13)	11.24 (10.45)	8.15 (13.18)	1.48 (17.72)	**<.001** ^a^

^a^ Significant differences with all cluster group comparisons.

^b^ Significant difference between clusters 1 and 2.

^c^ Significant difference between clusters 1 and 3.

^d^ Significant difference between clusters 2 and 3.

Abbreviations: BDI‐II, Beck Depression Inventory II;BMI, body mass index; CFT, Category Fluency Test; FAS, Functional Assessment Scale; MMSE, Mini‐Mental State Examination; PBA, Problem Behavior Assessment; PC, principal components.; SDMT, Symbol Digit Modalities Test; SIT, Stroop Interference Test; TFC, Total Functional Capacity; TMC, Total Maximum Chorea; UHDRS‐M, Unified Huntington's Disease Rating Scale‐Motor Assessment.

Bold values indicate significant differences among three clusters.

### 3.3 Brain MRI imaging features of the clusters

MRI data from a total of 28 HD and 17 HC were obtained. Of these, 7 patients were classified as cluster 1, 8 patients as cluster 2, and 13 patients as cluster 3 (Table 2). Compared to HC, the volumes of the entire brain and subcortical nuclei, including caudate nucleus, putamen, pallidum, and the cortical thickness were significantly reduced in HD (Table S2). There was a significant difference in whole brain volume between cluster 1 and cluster 2 or cluster 3 (1343.043 mm vs. 1464.451 mm, 1435.171 mm, respectively). In addition, the volume of the pallidum was significantly larger in cluster 2 than in cluster 1 or cluster 3 (0.363 cm^3^ vs. 0.327 cm^3^, 0.305 cm^3^, respectively) (Table 2).

**TABLE 2:** MRI imaging features in different clusters of HD.

MRI imaging features	Cluster 1	Cluster 2	Cluster 3	Total	*p* Value
*N*, %	7, 25.0%	8, 28.6%	13, 46.4%	28	NA
Whole brain volume (cm^3^), mean (SD)	1343.043 (83.415)	1464.451 (148.027)	1435.171 (143.961)	1420.505 (136.637)	**<.001** ^a^, ^b^
Caudate nucleus (cm^3^), mean (SD)	3.744 (1.608)	3.309 (1.871)	3.316 (0.784)	3.421 (1.338)	.906
Putamen (cm^3^), mean (SD)	5.290 (1.537)	5.199 (2.563)	4.846 (0.820)	5.058 (1.602)	.594
Pallidum (cm^3^), mean (SD)	0.327 (0.088)	0.363 (0.211)	0.305 (0.057)	0.327 (0.124)	**.017** ^b^, ^c^
Cortical thickness (mm), mean (SD)	2.191 (0.186)	2.184 (0.107)	2.220 (0.136)	2.202 (0.138)	.471

^a^ Significant difference between clusters 1 and 2.

^b^ Significant difference between clusters 1 and 3.

^c^ Significant difference between clusters 2 and 3.

Bold values indicate significant differences among three clusters.

### 3.4 The levels of plasma cytokines in the subtypes and in relation to the clinical features

**FIGURE 2:**
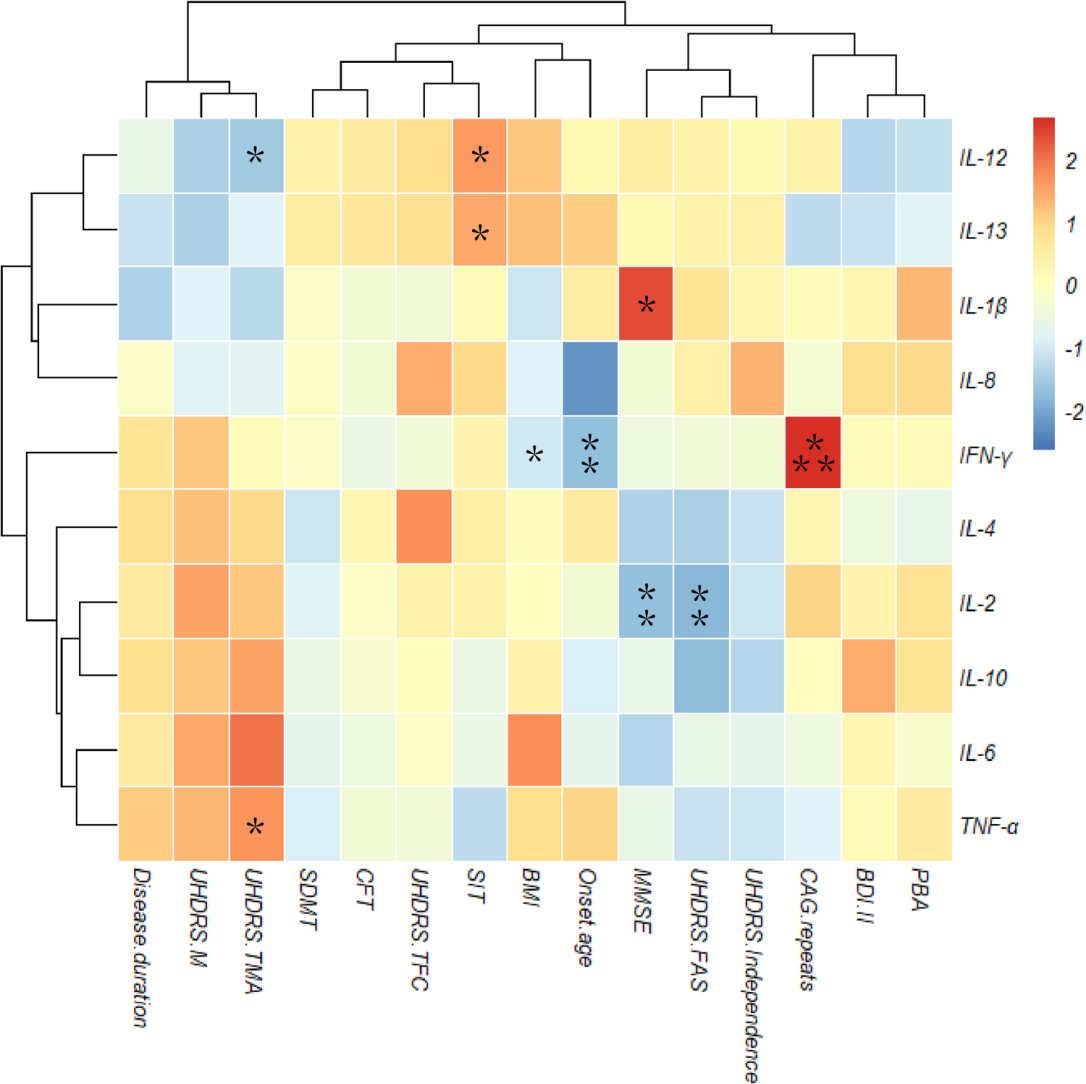
Correlations between cytokines and clinical features. The darker the red, the stronger the positive correlation, while the darker the blue, the stronger the negative correlation. *p < .05; **p < .01; ***p < .001.

A total of 28 HD patients (independent frm patients undergoing MRI) were tested for 10 cytokines. There were no significant differences in plasma cytokines levels among the three subtypes (Table S3, Figure S3). However, the plasma levels of IL‐2 were higher in cluster 1 compared to those in cluster 2 and cluster 3. Logistic regression analysis showed that IL‐2 was a determinator for cluster 1 (Table S4, OR = 3.074, *p* = .022). In addition, we found the plasma levels of IL‐2 were negatively correlated with UHDRS‐FAS (*r* = −.530) and MMSE (*r* = −.570), and the plasma levels of IFN‐γ were related to the number of *HTT* CAG repeats (*r* = .634), whereas patients with higher levels of plasma IFN‐γ tended to have early age of onset (*r* = −.555) (Table S5, Figure 2).

## 4 DISCUSSION

In this study, we established HD subtypes through a data‐driven analysis approach. The three subtypes classified in this study demonstrated distinct clinical features, neuroimaging patterns, and cytokines preferences. Patients in cluster 1 primarily exhibited a hypokinetic‐rigid type, characterized by both the longest CAG repeats and the most severe clinical symptoms, excluding chorea. Consistently, previous studies indicate that the number of CAG repeats is associated with disease severity, and associated with hypokinetic‐rigid subjects rather than choreatic subjects (McColgan & Tabrizi, 2018; Rosenblatt et al., 2006). Patients in cluster 2 exhibited the most severe chorea, but their overall motor symptoms were less severe than those in cluster 1. Cluster 2 also had benign mental and emotional problems, which align with findings from previous studies (Achenbach et al., 2020). This may be attributed to a higher degree of overactivation of the central dopaminergic pathway in cluster 2 (Schwab et al., 2015). Patients in cluster 3 presented with the most benign motor symptoms and cognitive function, but had moderate mental and emotional problems. Previous studies have shown that psychiatric problems are common symptoms in HD and may be obvious in some patients (Gubert et al., 2020; Walker, 2007).

Currently, there is limited research on the classification of clinical subtypes of HD based on motor phenotype, cognitive progression, dysarthric speech, and caregivers' evaluations (Table 3). Hart et al. previously subtyped HD according to motor phenotype, namely choreatic, hypokinetic‐rigid, and mixed types, and demonstrated that the progression of motor symptoms differed between predominantly hypokinetic‐rigid and predominantly choreatic subjects (Achenbach et al., 2020; Hart et al., 2013; Jacobs et al., 2016). Our results findings align with Hart et al.’s subtyping method (*p *= .004, Table 1). Using a two‐step k‐means cluster analysis model for cognitive analysis, HD has been divided into a slow cognitive progression group and an aggressive progression group (Martinez‐Horta et al., 2023). In regard to dysarthric speech of HD, HD has been divided into four subgroups (Diehl et al., 2019). Additionally, HD has been classified into four clusters based on different disease duration and caregivers’ burdens according to caregivers’ evaluation. In a study based on the European HD Network (EHDN) longitudinal cohort, HD patients were grouped into three clusters: rapid, moderate, and slow progressors (Ko et al., 2022). Compared to previous studies on HD subtyping, our study is the first to incorporate both motor and nonmotor symptoms of HD.

**TABLE 3:** Attempts to subtype HD.

*N*	Methods	Classifier input	Data used	Cluster features	References
1	Difference in total scores of UHDRS	UHDRS	European Huntington's Disease Network Registry study 1882 HD patients	Predominantly choreatic (28.1%), predominantly hypokinetic‐rigid types (22.9%), and mixed motor type (49.0%)	Hart et al., 2013
2	Two‐step k‐means cluster analysis	SDMT, SWRT, and MMSE	Enroll‐HD study 528 HD patients	Slow cognitive progression group (55.5%) and aggressive progression group (44.5%)	Martinez‐Horta et al., 2023
3	Unsupervised k‐means cluster analysis	Sentence Intelligibility Test, Rainbow Passage, and Mayo Clinic dysarthria rating scale	Vanderbilt University Medical Center 48 HD patients	Fast speaking rate, mild dysarthria (18.8%); slow speaking rate, moderate dysarthria (18.8%); slow speaking rate, mild dysarthria (33.3%); normal speaking rate mild dysarthria (29.1%)	Diehl et al., 2019
4	Unsupervised clustering analysis based on self‐organizing maps	Demographics, caregiver questionnaires and patients’ clinical characteristics	Departments from three French centers 148 caregivers and HD	A: advanced disease, irritability and obsessive‐compulsive behaviors, with high and increasing burden (29%); B: advanced disease, apathetic group with low and decreasing burden (21%); C: earlier stages with stable burden (26%); D: earlier stages with increasing burden due to depression (25%)	Youssov et al., 2022
5	Unsupervised k‐means cluster analysis	UHDRS, SWR, SDMT, and PBA	Enroll‐HD study 4961 HD patients	Rapid progressors (25.3%), moderate progressors (45.5%), and slow progressors (29.2%).	Ko et al., 2022
6	PCA and cluster analysis	Motor and nonmotor symptoms assessed by UHDRS, MMSE, SDMT, CFT, SIT, BDI‐II, and PBA	Tiantan Enroll‐HD study (104 participants)	Cluster 1 (27.7%) had the most severe motor and nonmotor symptoms; cluster 2 (31.3% had moderate motor symptoms and were characterized by most severe chorea; cluster 3 (41.0%) had the most benign motor symptoms but the most prominent nonmotor symptoms.	This study

Abbreviations: BDI‐II, Beck Depression Inventory II; CFT, Category Fluency Test; MMSE, Mini‐Mental State Examination; PBA, Problem Behavior Assessment; PCA, principal component analysis; SDMT, Symbol Digit Modalities Test; SIT, Stroop Interference Test; SIT, Stroop Interference Test; SWR, Stroop Word Reading; SWRT, Stroop Word‐Reading Test; UHDRS, Unified Huntington's Disease Rating Scale.

We further characterized different clusters based on the entire brain and basal ganglia volume, as well as cortical thickness using brain structural imaging. Our data are consistent with the fact that the whole brain atrophy in HD, especially in the basal ganglia, is more pronounced compared to those in controls (Kinnunen et al., 2021; Tabriz et al., 2009; Wijeratne et al., 2018). The volumes of the whole brain, caudate nucleus, putamen, and pallidum are associated with motor and nonmotor symptoms in HD, as well as disease duration (Kinnunen et al., 2021; Tabriz et al., 2009; Wijeratne et al., 2018). We found that cluster 1, which exhibited the worst performance in motor, mental, and cognition, had the lowest whole brain volume, indicating the importance of brain network connectivity for its proper function. Interestingly, patients in cluster 2 who had the most severe chorea, but the least mental problems, showed the largest pallidum volume after correction. A lower volume of the internal segment of the pallidum could increase its inhibitory action on the thalamus, reducing the thalamocortical neuronal excitatory input to the cortex and thereby contributing to hypokinesia (Singh‐Bains et al., 2016; Singh‐Bains et al., 2016). This could explain why cluster 2, with a larger volume of pallidum, tends to be associated with hyperkinesia and chorea. Pallidum is also an important part of pathways involved in depression, anxiety, and other mental problems (Correia et al., 2023; Liu et al., 2020; Sato et al., 2023; Wang et al., 2021). Preserving the volume of pallidum may be the reason for better psychiatric performance in cluster 2. The differences in brain structure imaging among the clusters support the rationale for this classification.

In this study, we found that IL‐2 was a crucial cytokine for distinguishing cluster 1. Elevated levels of IL‐2 were associated with poorer activity of daily living and cognitive function in cluster 1 patients. IL‐2 plays a pivotal role in maintaining immune homeostasis through its influence on regulatory cells and effector lymphocytes (Abbas et al., 2018). It is also linked to poorer clinical outcomes in other neurodegenerative diseases, although the underlying mechanism remains incompletely understood (Liu et al., 2022). Consistent with other studies, we found that plasma TNF‐α levels were associated with UHDRS chorea in HD (Bjorkqvist et al., 2008). TNF‐α is a proinflammatory cytokine that may be elevated by microglial activation and astrocytosis (Rocha et al., 2016). Furthermore, we observed a significant correlation between plasma levels of IFN‐γ and the number of CAG repetitions in *HTT*, indicating that HD is probably an autoimmune disease. IFN‐γ is a proinflammatory cytokine, and it may play a role in the initiation and progression of HD, analogous to its function in other neurodegenerative diseases (Glass et al., 2010; Kwon & Koh, 2020).

There are several limitations in this study. First, due to the rarity of HD, the sample size was relatively small, and not all enrolled participants underwent MRI imaging and cytokine testing. Second, this is a cross‐sectional study; the clinical presentations may vary during the course of the disease. Therefore, these findings should be validated in a larger longitudinal HD cohort with neuroimaging and cytokines data available.

## 5 CONCLUSIONS

In summary, we have delineated three clusters based on the demographic information, motor and nonmotor symptoms of HD patients. We have verified these clusters by their distinct clinical features, cytokine biomarkers, and neuroimaging markers. This is the first study subtyping HD using data‐driven approach and characterized further using different biomarkers. Our findings provide insights into the heterogeneous clinical presentations of HD, which may be used to improve clinical management and clinical trials for patients with HD. Meanwhile, our data suggested the pathophysiology of HD subtypes might be related to neuroinflammation as well as brain focal and network function.

## AUTHOR CONTRIBUTIONS

Ling‐Xiao Cao, Jin‐Hui Yin, Gang Du, and Qing Yang collected and analyzed the data. Ling‐Xiao Cao and Jin‐Hui Yin wrote the first draft. Yue Huang designed and supervised the study.

## CONFLICT OF INTEREST STATEMENT

The authors declare no conflict of interest.

### PATIENT CONSENT STATEMENT

All participants agreed to participate in this study and signed an informed consent form before enrollment.

### PEER REVIEW

The peer review history for this article is available at https://publons.com/publon/10.1002/brb3.3469.

## Supporting information

Figure S1. Selection of optimal cluster number through elbow plots (A) and gap statistic (B).

Figure S2. Comparison of clinical differences among three clusters. The darker the red color, the more it exceeds the mean, while the darker the blue color, the more it falls below the mean. ^a^Significant differences with all cluster group comparisons. ^b^Significant difference between clusters 1 and 2. ^c^Significant difference between clusters 1 and 3. ^d^Significant difference between clusters 2 and 3.

Figure S3. Comparison of cytokines among three clusters. ^a^Significant difference between clusters 1 and 2.

Table S1. Demographic and clinical characteristics of the participants.
Table S2. MRI imaging features in HD and controls.
Table S3. Cytokine levels in different clusters of HD.
Table S4. Associations between cytokines and clusters.
Table S5. Correlation between cytokines and clinical features in HD

## Data Availability

The data that support the findings of this study are available from the corresponding author upon reasonable request.
