# Supplementary figures and images for: Identifying and verifying Huntington's disease subtypes: Clinical features, neuroimaging, and cytokine changes
Source: Brain Behav. 2024 Mar 17;14(3):e3469. doi: 10.1002/brb3.3469 (PMC10945031; doi:10.1002/brb3.3469)

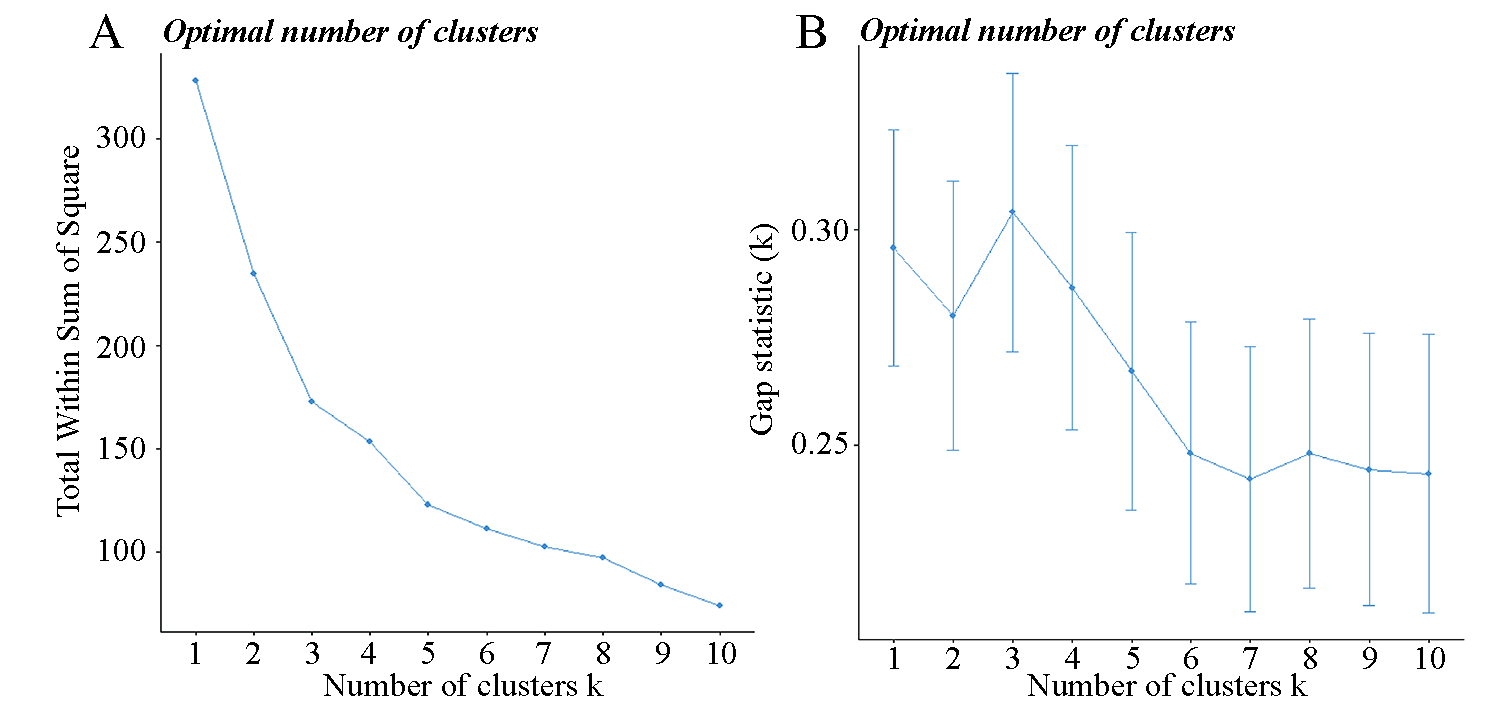

Supplement: Supplementary file 1 — Figure S1. Selection of optimal cluster number through elbow plots (A) and gap statistic (B). [file BRB3-14-e3469-s003.tif]

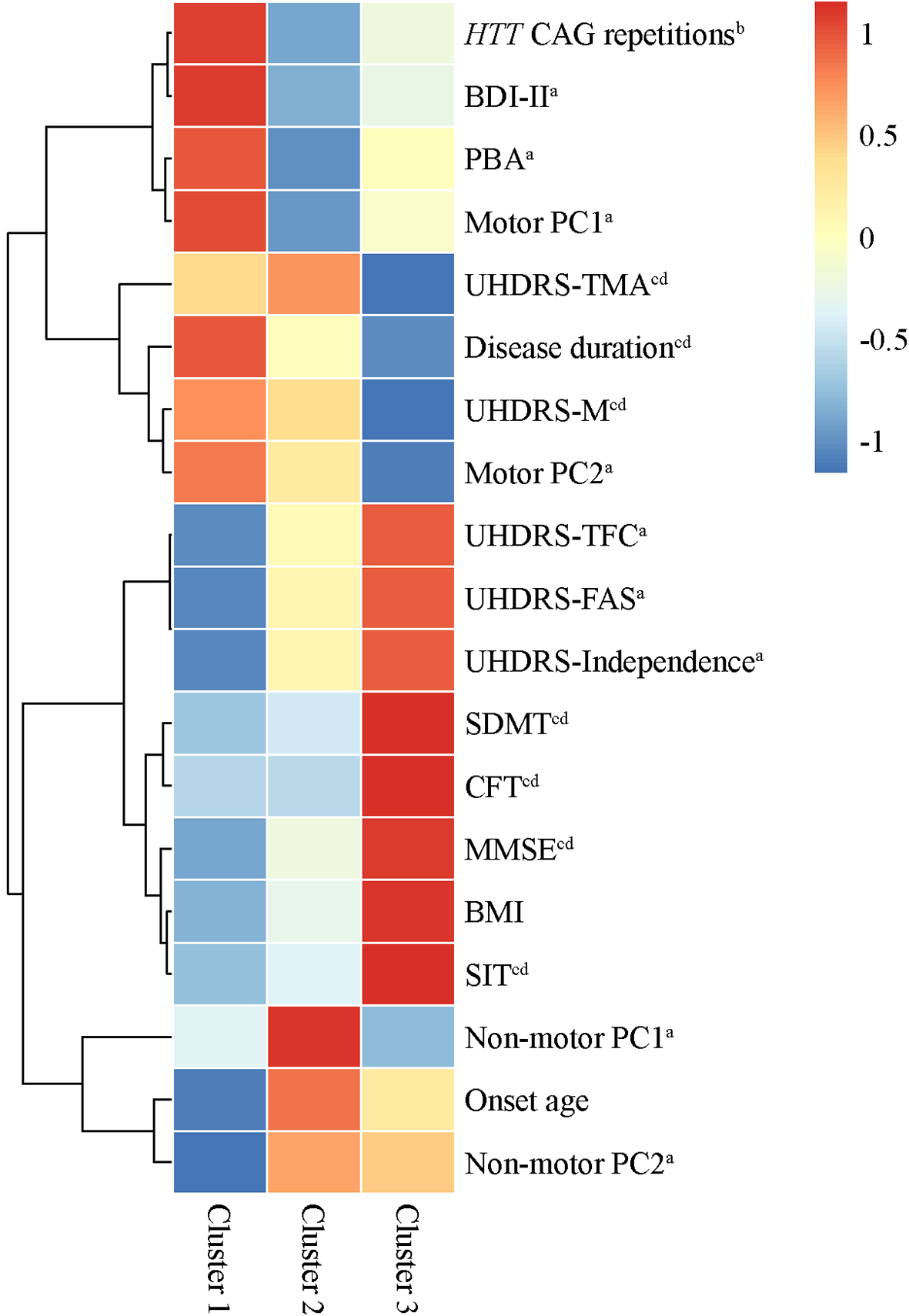

Supplement: Supplementary file 2 — Figure S2. Comparison of clinical differences among three clusters. The darker the red color, the more it exceeds the mean, while the darker the blue color, the more it falls below the mean. aSignificant differences with all cluster group comparisons. bSignificant difference between clusters 1 and 2. cSignificant difference between clusters 1 and 3. dSignificant difference between clusters 2 and 3. [file BRB3-14-e3469-s002.tif]

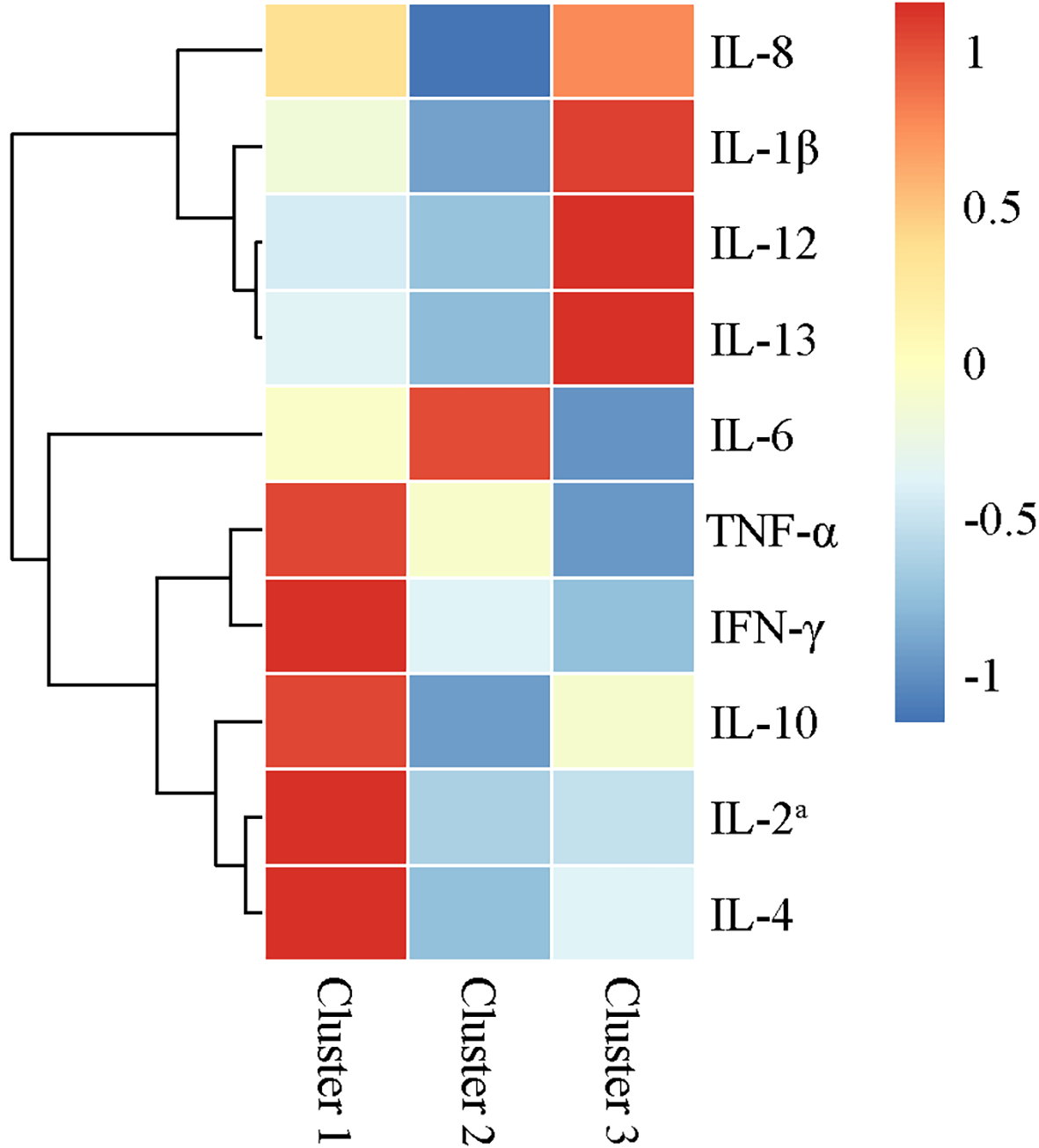

Supplement: Supplementary file 3 — Figure S3. Comparison of cytokines among three clusters. aSignificant difference between clusters 1 and 2. [file BRB3-14-e3469-s001.tif]
