# Supplementary material for: Identifying and verifying Huntington's disease subtypes: Clinical features, neuroimaging, and cytokine changes
Source: Brain Behav. 2024 Mar 17;14(3):e3469. doi: 10.1002/brb3.3469 (PMC10945031; doi:10.1002/brb3.3469)
Supplement: Supplementary file 4 — Table S1. Demographic and clinical characteristics of the participants. Table S2. MRI imaging features in HD and controls. Table S3. Cytokine levels in different clusters of HD. Table S4. Associations between cytokines and clusters. Table S5. Correlation between cytokines and clinical features in HD [file BRB3-14-e3469-s004.docx]

**Supplementary Table 1. Demographic and Clinical Characteristics of the participants**

| **Clinical characteristics** | **HD (N=83)** | **Pre-HD (N=21)** | **HC (N=31)** | ***P* value** |
| --- | --- | --- | --- | --- |
| Gender, male (%) | 41(49.4%) | 9 (42.9%) | 10(32.3%) | NA |
| Onset age | 42.94(11.77) | NA | NA | NA |
| Disease duration | 4.66(3.11) | NA | NA | NA |
| HTT CAG repetitions | 45.78(7.05) | 43.76(3.18) | NA | 0.204 |
| UHDRS-M | 47.67(25.42) | 0.52(1.44) | NA | **<0.001** ^b^ |
| UHDRS-TMC | 11.22(6.85) | 0.10(0.30) | NA | **<0.001** ^b^ |
| UHDRS-FAS | 18.47(5.92) | 24.67(0.58) | NA | **<0.001** ^b^ |
| UHDRS-Independence | 79.34(18.16) | 100(0.00) | NA | **<0.001** ^b^ |
| UHDRS-TFC | 8.10(4.00) | 13.05(0.22) | NA | **<0.001** ^b^ |
| SIT | 18.35(9.29) | 40.81(12.25) | 34.97(11.44) | **<0.001** ^a^ |
| SDMT | 19.05(9.86) | 50.81(11.02) | 46.77(13.52) | **<0.001** ^bc^ |
| CFT | 10.35(5.42) | 19.67(6.83) | 19.52(4.70) | **<0.001** ^bc^ |
| BDI-II | 11.76(9.76) | 6.29(9.05) | 5.06(7.72) | **<0.001** ^bc^ |
| MMSE | 23.34(5.26) | 28.90(1.73) | 29.23(1.33) | **<0.001** ^bc^ |
| PBA | 21.30(16.65) | 11.38(11.77) | NA | **0.012** ^b^ |

^a^ Significant difference between HD, preHD, and HC; ^b^ Significant difference between HD and preHD; ^c^ Significant difference between HD and HC.

Abbreviations: HD, Huntington's disease; pre-HD, premanifest HD; HC, health control; UHDRS-M, Unified Huntington’s Disease Rating Scale-Motor Assessment; TMC, Total Maximum Chorea; FAS, Functional Assessment Scale; TFC, Total Functional Capacity; MMSE, Mini-Mental State Examination; SDMT, Symbol Digit Modalities Test; CFT, Category Fluency Test; SIT, Stroop Interference Test; BDI-II, Beck Depression Inventory II; PBA, Problem Behavior Assessment.

**Supplementary Table 2. MRI imaging features in HD and controls**

| **MRI Imaging features** | **HD** | **HC** | ***P* value** |
| --- | --- | --- | --- |
| N (male %) | 28(12, 42.9%) | 17(7, 41.2%) | NA |
| Age at examination (years old), mean (SD) | 48.250(13.012) | 47.176(12.043) | 0.784 |
| Whole brain volume (cm^3^), mean(SD) | 1420.505(136.637) | 1451.033(141.428) | **<0.001** |
| Caudate nucleus (cm^3^), mean(SD) | 3.421(1.338) | 6.639(0.914) | **0.001** |
| Putamen (cm^3^), mean(SD) | 5.058(1.602) | 8.435(0.623) | **0.001** |
| Pallidum (cm^3^), mean(SD) | 0.327(0.124) | 0.618(0.106) | **0.004** |
| Cortical thickness (mm), mean(SD) | 2.202(0.138) | 2.377(0.085) | **0.006** |

**Supplementary Table 3. Cytokine levels in different clusters of HD**

|  | **Cluster 1** | **Cluster 2** | **Cluster 3** | **Total** | ***P* value** |
| --- | --- | --- | --- | --- | --- |
| N | 8, 28.6% | 10, 35.7% | 10, 35.7% | 28 | NA |
| IL-1β | 0.028(0.029) | 0.025(0.021) | 0.033(0.026) | 0.029(0.025) | 0.796 |
| **IL-2** | **0.225(0.153)** | **0.115(0.079)** | **0.121(0.084)** | **0.148(0.114)** | **0.075 ^a^** |
| IL-4 | 0.010(0.011) | 0.005(0.006) | 0.006(0.004) | 0.007(0.007) | 0.396 |
| IL-6 | 0.473(0.207) | 0.519(0.248) | 0.433(0.167) | 0.475(0.206) | 0.662 |
| IL-8 | 2.800(1.134) | 2.359(1.086) | 2.921(1.275) | 2.686(1.154) | 0.541 |
| IL-10 | 0.181(0.104) | 0.109(0.042) | 0.139(0.070) | 0.140(0.077) | 0.139 |
| IL-12p70 | 0.088(0.079) | 0.078(0.051) | 0.142(0.188) | 0.103(0.123) | 0.478 |
| IL-13 | 0.354(0.299) | 0.314(0.212) | 0.502(0.540) | 0.393(0.378) | 0.528 |
| TNF-α | 3.542(0.954) | 3.300(0.617) | 3.116(1.014) | 3.303(0.858) | 0.596 |
| IFN-γ | 10.899(7.459) | 8.206(5.770) | 7.561(4.721) | 8.745(5.912) | 0.478 |

^a^ Significant difference between cluster 1 and 2

**Supplementary Table 4. Associations between cytokines and clusters**

| **Cytokines** | **Cluster 1** | | **Cluster 2** | | **Cluster 3** | |
| --- | --- | --- | --- | --- | --- | --- |
|  | **OR** | ***P* value** | **OR** | ***P* value** | **OR** | ***P* value** |
| IL-1β | 1.375 | 0.973 | 0.684 | 0.560 | 0.880 | 0.537 |
| IL-2 | **3.074** | **0.022** | 0.421 | 0.254 | 0.313 | 0.355 |
| IL-4 | 4.875 | 0.207 | 0.814 | 0.416 | 1.517 | 0.653 |
| IL-6 | 0.193 | 0.979 | 9.556 | 0.413 | 0.821 | 0.428 |
| IL-8 | 1.101 | 0.760 | 0.330 | 0.276 | 2.346 | 0.433 |
| IL-10 | 7.547 | 0.100 | 0.162 | 0.118 | 1.948 | 0.937 |
| IL-12p70 | 0.007 | 0.696 | 3.262 | 0.417 | 15.482 | 0.229 |
| IL-13 | 6.204 | 0.755 | 0.200 | 0.424 | 0.967 | 0.267 |
| TNF-α | 3.156 | 0.392 | 2.313 | 0.987 | 0.127 | 0.401 |
| IFN-γ | 10.266 | 0.262 | 0.684 | 0.726 | 0.356 | 0.441 |

**Supplementary Table 5. Correlation between cytokines and clinical features in HD**

| **Cytokines** | **CAG repeats** | **BMI** | **Onset age** | **Disease duration** | **UHDRS-M** | **TMC** | **FAS** | **Independence** | **TFC** | **SIT** | **SDMT** | **CFT** | **BDI-II** | **MMSE** | **PBA** |
| --- | --- | --- | --- | --- | --- | --- | --- | --- | --- | --- | --- | --- | --- | --- | --- |
| **IL-1β** | 0.041 | -0.143 | 0.102 | -0.198 | -0.114 | -0.176 | 0.140 | 0.062 | -0.041 | 0.050 | 0.003 | -0.031 | 0.059 | **0.398^*^** | 0.227 |
| **IL-2** | 0.176 | -0.078 | -0.161 | 0.069 | 0.314 | 0.216 | **-0.530^**^** | -0.340 | 0.018 | 0.018 | -0.270 | -0.085 | 0.008 | **-0.507^**^** | 0.119 |
| **IL-4** | -0.007 | -0.032 | 0.054 | 0.103 | 0.164 | 0.120 | -0.294 | -0.243 | 0.258 | 0.035 | -0.230 | -0.007 | -0.134 | -0.288 | -0.161 |
| **IL-6** | -0.141 | 0.278 | -0.180 | 0.054 | 0.226 | 0.329 | -0.165 | -0.181 | -0.061 | -0.154 | -0.181 | -0.143 | -0.002 | -0.301 | -0.079 |
| **IL-8** | -0.008 | -0.074 | -0.237 | 0.010 | -0.073 | -0.065 | 0.073 | 0.178 | 0.190 | 0.135 | 0.016 | -0.016 | 0.125 | -0.012 | 0.130 |
| **IL-10** | 0.021 | 0.100 | -0.171 | 0.211 | 0.264 | 0.348 | -0.347 | -0.260 | 0.025 | -0.092 | -0.092 | -0.027 | 0.327 | -0.119 | 0.186 |
| **IL-12p70** | 0.109 | 0.306 | 0.066 | -0.132 | -0.342 | **-0.375^*^** | 0.105 | 0.065 | 0.233 | **0.421^*^** | 0.103 | 0.162 | -0.322 | 0.139 | -0.286 |
| **IL-13** | -0.250 | 0.311 | 0.283 | -0.223 | -0.302 | -0.166 | 0.122 | 0.134 | 0.224 | **0.384^*^** | 0.155 | 0.198 | -0.221 | 0.084 | -0.154 |
| **TNF-α** | -0.220 | 0.207 | 0.231 | 0.258 | 0.300 | **0.399^*^** | -0.273 | -0.269 | -0.097 | -0.298 | -0.224 | -0.091 | 0.023 | -0.151 | 0.128 |
| **IFN-γ** | **0.634^***^** | **-0.375^*^** | **-0.555^**^** | 0.108 | 0.224 | -0.065 | -0.196 | -0.189 | -0.191 | -0.015 | -0.125 | -0.239 | -0.070 | -0.220 | -0.054 |

* *p*<0.05; ** *p*<0.01; *** *p*<0.001.

Abbreviations: BMI, body mass index; UHDRS-M, Unified Huntington’s Disease Rating Scale-Motor Assessment; TMC, Total Maximum Chorea; FAS, Functional Assessment Scale; TFC, Total Functional Capacity; MMSE, Mini-Mental State Examination; SDMT, Symbol Digit Modalities Test; CFT, Category Fluency Test; SIT, Stroop Interference Test; BDI-II, Beck Depression Inventory II; PBA, Problem Behavior Assessment.
